# Memory decline in older individuals predicts an objective indicator of oral health: findings from the Sydney Memory and Ageing Study

**DOI:** 10.1186/s12903-022-02128-y

**Published:** 2022-03-27

**Authors:** Nithin Manchery, Julie D. Henry, Ben C. P. Lam, Nicole A. Kochan, Alan Deutsch, Henry Brodaty, Perminder S.Sachdev, Matthew R. Nangle

**Affiliations:** 1 School of Dentistry, The University of Queensland, Brisbane, QLD 4006 Australia; 2 School of Psychology, The University of Queensland, Brisbane, QLD Australia; 3 Centre for Healthy Brain Ageing, School of Psychiatry, Faculty of Medicine, University of New South Wales, Sydney, Australia; 4 Centre for Education and Research On Ageing, Concord Hospital, University of Sydney, Sydney, NSW Australia

**Keywords:** Cognitive function, Cognitive decline, Oral health deterioration, Salivary pH

## Abstract

**Background:**

Growing evidence suggests that there is an association between poor oral health and cognitive function in late adulthood. However, most studies to date have relied on cross-sectional research methods that do not permit inferences about the temporality of any association. Moreover, the few longitudinal studies that do exist have typically relied on small samples and quite limited cognitive or oral health assessments. The aim of the present study was therefore designed to provide the first direct evaluation of whether cognitive function is predictive of poor oral health in older adults.

**Methods:**

This longitudinal research included data from 339 participants aged 70 years or older from The Sydney Memory and Ageing Study (MAS), a large cohort of healthy community-dwelling older adults. Cognitive function was assessed using a battery of tests at baseline (Wave 1) in 2005 and six years later (Wave 4) in 2011. In 2015 (Wave 6), participants were assessed for oral health using the Oral Health Assessment Tool (OHAT), number of functional occluding pairs of natural teeth and sublingual resting saliva pH (SRSpH). Ordinal least squares regression analysis was used to model the effect of cognitive function on total OHAT score, and binomial logistic regression used for SRSpH and occluding pairs of functional teeth.

**Results:**

Two models were tested. In the partially adjusted model, age, gender and years of education were included. The fully adjusted model additionally included medical conditions, general health, depression, smoking, alcohol consumption, functionality, and dental care utilization. The key finding to emerge was that a six-year change in memory (from Wave 1 to Wave 4) was associated with lower sublingual resting saliva pH at Wave 6 in partially (Odds Ratio (OR) = 0.65) and fully adjusted model (OR = 0.63).

**Conclusions:**

This longitudinal study provides further evidence that a relationship between cognitive function and oral health exists, and also points to this relationship potentially being bi-directional, as previous evidence suggests. The findings from the study also suggest that older adults who present with greater than normal memory decline at an earlier point in life were more likely to experience poor oral health when this was evaluated at a later time-point, four years later.

**Supplementary Information:**

The online version contains supplementary material available at 10.1186/s12903-022-02128-y.

## Background

The proportion of older adults worldwide is increasing and projected to continue to do so. In 2019, the number of people globally aged 65 or over was estimated to be 703 million, and this is expected to double to 1.5 billion by 2050 [1]. This change in the ageing demographic is important for several reasons, not least because older age remains the single most important predictor of cognitive impairment, which is the leading cause of dependence and disability among elderly adults [2]. Dementia, which is the pathological extremity of cognitive impairment affects more than 50 million people worldwide, and is a major public health burden [3].

The importance of oral health is often not fully appreciated by health care professionals working in aged care, yet oral health is fundamental to older adults’ general health, overall well-being and quality of life [4]. Poor oral health directly impacts older adults’ physical wellbeing, by affecting their ability to eat and swallow properly, contributing to weight loss, dehydration, malnutrition, and frailty [5], and can also negatively impact social communication [6]. Periodontal disease severity and Porphyromonas gingivalis have also been significantly associated with all-cause mortality in cardiovascular disease and ischemic stroke [7]. Further, both Porphyromonas gingivalis and its proteases, involved in periodontal disease, have been identified in the brains of Alzheimer’s patients and have been identified as one of many potential risk factors for the development of this disorder [8].

Relative to younger adults, older people are more vulnerable to a range of oral health problems [9]. A range of potential risk factors have been studied, including reduced functional capacity, decreased physical strength, the presence of comorbid medical conditions, polypharmacy and of particular interest in recent years, cognitive impairment [10, 11]. A recent systematic review concluded that there was moderate to good evidence for an association between three specific cognitive domains (learning and memory, complex attention and executive function) and older adults’ oral health [12]. However, this review also highlighted an important limitation of this literature, noting that most studies conducted to date that have tested this relationship have relied on cross-sectional research methods. Such methods do not allow inferences to be made about the temporality of any association and, consequently, causality.

Interestingly, both potential directions of causality appear viable. This is because, on the one hand, it has been suggested that poorer oral health may contribute to cognitive decline via specific biological mechanisms such as common inflammatory processes linked to periodontitis [13] or reduced masticatory performance leading to reduced nutritional intake [14]. With respect to the reverse, it has been suggested that cognitive decline might lead to oral health deterioration as functionality in daily life decreases, via reduced attention to oral hygiene or inadequate use of dental health services.

However, surprisingly few studies have used longitudinal research methods to directly test whether there is evidence to support this latter causal pathway. Although it has been shown that older adults with poorer cognitive functioning are less likely to access dental care [15], and engage in less frequent tooth brushing and related oral hygiene practices [16, 17], only six studies have directly tested whether cognitive function at an earlier time point predicts oral health at a later one [18–23]. Moreover, inferences from these studies are limited by the use of small samples [19, 21], and in five of the studies, reliance on only brief cognitive screens or medical records to quantify cognitive function [18–22]. While the sixth study included a large community cohort, and excellent characterisation of cognitive function [23], a limitation here was that oral health was indexed solely via self-report. Extensive literature shows that self-report measures of oral health are less accurate than more objective indices [24, 25].

Thus, although Kang et al. [23] found that lower cognitive function at baseline was associated with poorer oral health at follow up, the next important step in this literature is to establish these associations using objective indicators of oral health. The present study was therefore designed to provide the first direct evaluation of whether cognitive function is predictive of poor oral health in older adults, using a battery of validated, objective assessments to index both cognitive function and oral health. The key predictions were that older adults’ cognitive function at Wave 4, and degree of subsequent cognitive decline (from Wave 1 to Wave 4), would be predictive of their subsequent oral health, even after adjusting for important potential covariates.

## Methods

### Study population

Participants were drawn from the community-based Sydney Memory and Aging (MAS) study. The MAS is an ongoing, longitudinal study that originally recruited 1037 healthy individuals aged 70–90 years [26]. MAS participants have completed an extensive range of physical, cognitive and psychological assessments; at study entry commencing in 2005 (Wave 1), and follow-up assessments approximately every 2 years thereafter. To be eligible for inclusion into MAS, participants had to be free from any major neurological or psychiatric illness including a self-reported history of dementia, and also pass a cognitive screen to rule out dementia at study entry. Participants with a Mini Mental State Examination (MMSE) score of < 24 adjusted for age, education and non-English speaking background were excluded.

**Fig. 1:**
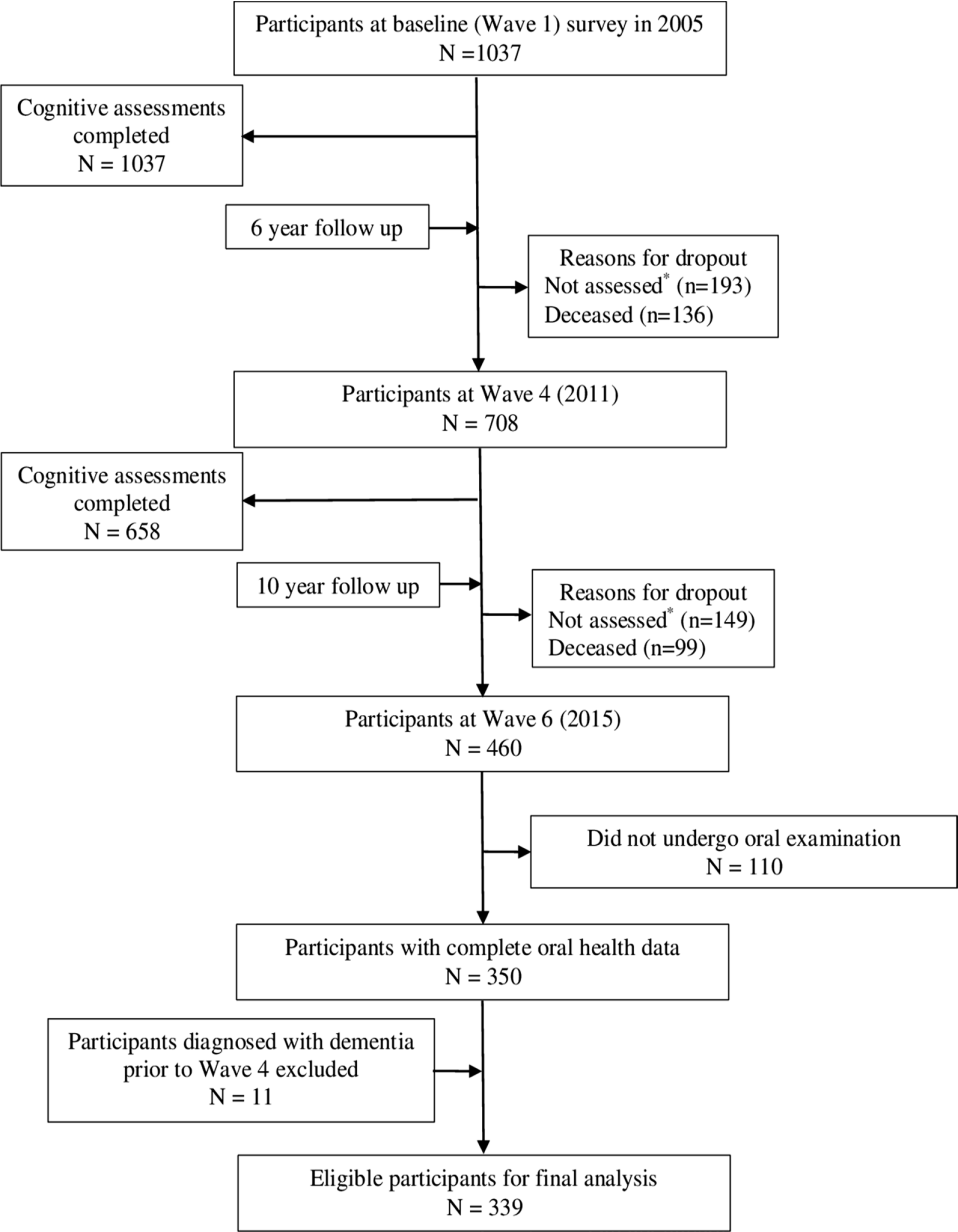
Flowchart for participant selection for this study

In the present study, analyses were restricted to participants who completed an oral health assessment ten years after baseline at Wave 6 (2015) and had not been diagnosed with dementia at any time point up to Wave 4 (2011). At Wave 4, the total sample included 708 participants. Of these, 460 participants then went on to complete Wave 6, of whom 350 undertook the oral health assessments. After excluding the 11 participants who were diagnosed with dementia prior to Wave 4, the final sample consisted of 339 participants (Fig. 1). Cognitive data in the study was obtained from Wave 1 (2005) and Wave 4 (2011) respectively, and oral health data available only at Wave 6 (2015).

### Oral health

The well validated Oral Health Assessment Tool (OHAT) [27] was used for assessing oral health at Wave 6. The OHAT assesses the following aspects of oral health: (i) lips; (ii) tongue; (iii) gums and oral tissues; (iv) saliva; (v) natural teeth; (vi) dentures; (vii) oral cleanliness; and (viii) dental pain. To administer OHAT, trained research assistants inspected the oral cavity for 5–10 min. Scores on individual categories of OHAT range from healthy (0), changes from normal (1) to unhealthy (2). Sum of scores from each category was derived, giving a total score ranging from 0 (healthy) to 16 (extensive oral health problems), with higher OHAT scores indicative of poorer oral health.

Other oral health measures recorded were sublingual resting saliva pH (SRSpH) and the number of functional occluding pairs of natural teeth. Saliva was obtained from beneath participants’ tongue using a micro brush for two seconds, and rubbed on one square of 6–8 pH paper and one square of 3–5.5 pH paper. All the included participants were instructed not to eat or drink 1 h prior to the saliva test, and any related abnormalities thought to be associated with salivary pH reduction not excluded. The test paper was observed for whether the micro brush could wet the pH paper and for colour change and salivary pH levels recorded. A lower pH score was recorded in cases where the level appeared to be between two values [28]. All numbers equal to 6.6 or above were considered normal, while values lower than 6.6 were considered as greater risk for oral disease [29]. Healthy salivary pH should measure no lower than 6.6, and lower salivary pH is well-established as a risk factor for dental caries and dental erosion [30]. The number of occluding pairs (maximum of 16 pairs) was recorded by asking the participant to bite in maximum intercuspation, which was then counted objectively. This was categorized into (i) > 10 pairs, and (ii) ≤ 10 pairs of functional teeth [31].

### Cognitive function

A battery of standardized cognitive assessments was used to assess cognitive function at waves 1 to 4 (neuropsychological battery was available only for waves 1–4 but not in other waves). Memory was measured using Logical Memory Story A (delayed) [32], Rey Auditory Visual Verbal Learning Test (RAVLT) [33] (total learning; trials 1–5; short-term recall: trial 6, and long-term recall: trial 7) and the Benton Visual Retention Test (BVRT) [34]. Executive function was assessed using Phonemic Fluency (FAS) [35] and the Trail Making Test B [33]. To assess attention/processing speed, Digit Symbol Coding [32] and Trail Making Test A [33] were used. Animal naming [36] and the 30-item Boston Naming Test [37] were used to assess Language. Visuospatial ability was measured using the Block Design test [38]. Raw test scores were transformed to z-scores using the baseline mean and standard deviation (SD) values of a healthy reference subsample (*n* = 723 MAS participants) [39]. Domain scores were calculated by averaging the z-scores of component tests (except for visuo-spatial which was represented by a single test), again standardized by transforming these scores using the healthy group baseline mean and SD values. Global cognition scores were calculated by averaging individual cognitive domain scores (attention/processing speed, memory, language, executive function and visuo-spatial) and standardized against the healthy group.

### Covariates

Established or suspected factors associated with dental status and cognitive function were selected based on prior literature. Socio-demographic characteristics included age, gender and years of education. Medical conditions were defined as history of stroke, diabetes, hypertension, heart disease (coronary heart disease, atrial fibrillation) [40] and lung disease (chronic obstructive pulmonary disease) up to Wave 4. Self-rated general health status was assessed on a Likert scale with anchors 1 = poor to 5 = excellent. The shorter version of Geriatric Depression Scale (GDS), consisting of 15 questions, was used to evaluate depressive symptoms [41]. Of the 15 items, 10 indicated the presence of depression when answered positively, while the rest (question numbers 1, 5, 7, 11, 13) indicated depression when answered negatively. The total score was calculated by totaling 1 point counted for each depression answer. The scores on the GDS can range from 0–15. Health related behaviours included smoking patterns (responses categorized into never, past and current smokers) and alcohol consumption (categorized as– abstainer (0 drinks), <  = 1 standard drink/day, or > 1 standard drink/day – with 1 standard drink = 10 g alcohol) [40]. Functional impairment was defined as informant-reported limitations on Bayer activities of daily living (B-ADL) [42] and basic activities of daily living (ADL) [43]. The basic ADL was assessed using the Lawton & Brody Physical Self-Maintenance Scale (PSMS) [43]. The PSMS is a scale containing 6 items of self-care with a 5-point rating scale (responses ranging from total independence to total dependence). For each item the response describing the person’s highest level of functioning is recorded (either 1 or 0). The total score on the basic ADL ranges from 0 to 6. The instrumental ADL was assessed using the Bayer-Activities of Daily Living Scale (B-ADL) [42]. The informant rates a person’s ability to perform an activity on a scale of 1 to 10 for each of the 25 items, where 1 indicates ‘never’ and 10 indicates ‘always’ have difficulty. In instance where an activity is not appropriate, not relevant, or unknown, the informant rates ‘not applicable’ or ‘unknown’. Individual item scores are then summed up. Items rated ‘not applicable’ and ‘unknown’ are not used for the computation of the total scores. The total is then divided by the number of items rates between ‘1’ and ‘10’. The total scores on B-ADL range between 1.00 and 10.00. Dental care utilization, measured at Wave 6, was based on a question asking when participant last saw a dentist (where 1 =  < 12 months ago, 2 = 1–2 years ago, 3 = 3–4 years ago, 4 = 5–6 years ago, and 5 =  > 6 years ago).

### Statistical analysis

All statistical tests were 2-sided, and significance was set at *p* ≤ 0.05. Statistical analyses were performed using IBM SPSS Statistical software for Windows version 27.0. Ordinal least squares regression was used for modelling the effect of cognitive function on total OHAT score. Binomial logistic regression was used for the dichotomized categorical variables (SRSpH and pairs of functional teeth). Two sets of models were tested. In the partially adjusted model, age, gender and years of education were included. The fully adjusted model included covariates in the partially adjusted model plus medical conditions, general health, depression, smoking, alcohol consumption, functionality, and dental care utilization. Multiple imputations by chained equation were used to impute missing covariate data. Twenty imputed datasets were created and analysed, and results were pooled based on Rubin’s rules. The effects of cognitive function at Wave 4 and change in cognitive function from Wave 1 to Wave 4 were examined in separate sets of models. To capture the change in cognition, change scores were computed by subtracting Wave 1 cognitive scores from Wave 4 cognitive scores.

## Results

### Participant characteristics

Table 1 summarizes detailed characteristics of all participants from the MAS study. The age of the participants at study entry ranged from 70–87 years, and females were slightly over-represented (55%). The participants selected were relatively younger, more likely to be female, less likely to have coronary artery disease, had fewer depressive symptoms, had better self-rated general health and basic ADL scores, and were more likely to be non-smokers, relative to the unselected participants. In addition, of the participants selected, 303 (89%) reported their perceived general health as being good to excellent. The most prevalent condition reported in the medical history was hypertension (59%). About half of the included participants were ex-smokers (47%) and consumed alcohol (50%) on a daily/regular basis. Over a median interval of six years between Wave 1 and Wave 4, participants experienced a decline in cognitive function. The magnitude of the effect/change for the cognitive tests ranged from small (for assessments of memory) to medium (for global cognition) (see Additional file 1).

**Table 1:** Baseline characteristics of participants in the whole MAS sample (N = 1037), selected sample (N = 339) and unselected sample (N = 698)

	Whole^a^ sample (N = 1037)	Selected^b^ sample (N = 339)	Unselected^c^ sample (N = 698)	Difference between selected and unselected
Variables	Mean (SD)/n(%)	Mean (SD)/n(%)	Mean (SD)/n(%)	*p* value^#^
Age	78.3 (4.8)	76.1 (3.9)	79.4 (4.8)	< .001
Female	572 (55.1%)	203 (59.8%)	369 (52.8%)	.033
Education in years	11.6 (3.4)	11.8 (3.4)	11.4 (3.5)	.086
MMSE	28.7 (1.3)	28.8 (1.3)	28.7 (1.4)	.185
Medical conditions				
Ever had stroke	41 (3.9%)	9 (2.6%)	32 (4.6%)	.139
Ever had diabetes	126 (12.2%)	32 (9.4%)	94 (13.5%)	.057
Ever had hypertension	629 (60.8%)	200 (59.3%)	429 (61.6%)	.479
Heart diseases				
* Ever had CAD*	198 (19.0%)	48 (14.1%)	150 (21.4%)	.005
* Ever had AF*	69 (6.7%)	17 (5.1%)	52 (7.5%)	.146
Lung disease				
* Ever had COPD*	20 (1.9%)	4 (1.1%)	16 (2.2%)	.222
General health				< .001
Excellent	104 (10.0%)	38 (11.2%)	66 (9.4%)	
Very good	323 (31.2%)	127 (37.5%)	196 (28.1%)	
Good	436 (42.1%)	138 (40.8%)	298 (42.8%)	
Fair	154 (14.8%)	33 (9.7%)	121 (17.3%)	
Poor	17 (1.6%)	2 (0.5%)	15 (2.1%)	
GDS	2.2 (2.0)	1.8 (1.7)	2.5 (2.1)	< .001
Smoking status				< .001
Current smoker	40 (4.5%)	11 (3.2%)	29 (5.3%)	
Past smoker	514 (58.4%)	160 (47.2%)	354 (65.5%)	
Never smoker	325 (36.9%)	168 (49.5%)	157 (29.0%)	
Alcohol consumption				.965
Abstainer (0 drinks)	130 (12.5%)	43 (12.6%)	87 (12.4%)	
≤ 1 standard drink per day	391 (37.7%)	126 (37.1%)	265 (38.0%)	
> 1 standard drink	515 (49.7%)	170 (50.1%)	345 (49.5%)	
Functional impairment				
IADL	14 (2.0%)	5 (2.2%)	9 (1.9%)	.753
ADL	92 (9.4%)	17 (5.1%)	75 (11.6%)	.001

*n* number of participants, *SD* Standard Deviation**,** *MMSE* Mini Mental State Examination, *CAD* Coronary Artery, Disease, *AF* Atrial Fibrillation, *COPD* Chronic Obstructive Pulmonary Disease, *GDS* Geriatric Depression Scale, *IADL* Instrumental Activities of Daily Living, *ADL* Activities of Daily Living

^a^Refers to all participants at baseline (Wave 1)

^b^Refers to only those participants included in the present study for analysis

^c^Refers to excluded participants in the study (e.g., those not followed up, people who developed dementia, people who did not have oral health data)

^**#**^Independent *t-test* or Mann–Whitney U-test (if skewed) was conducted for continuous variables. Chi-square-difference test was conducted for categorical variables

*p ≤ .05, **p < .01, ***p < .001

### Oral health characteristics

Scores on individual distribution categories for OHAT is reported in Table 2. The average OHAT score was 2.5 (range = 0–11), and mean SRSpH ranged from 4.5 to 7.8 (M = 6.1, SD = 0.5). More than half of the participants had 20 or more occluding pairs of teeth (62%), and had visited the dentist in the last 12 months (68%). Taken together, the relatively low scores on OHAT, the relatively high number of functional pairs of teeth, and frequent dentist visits indicate that majority of participants in this study were generally reporting relatively good levels of oral health (Table 3).

**Table 2:** Distribution of scores for individual categories of the Oral Health Assessment Tool (OHAT) for participants at Wave 6 (N = 339)

Category*	Score 0	Score 1	Score 2
	*n* (%)	*n* (%)	*n* (%)
Lips	285 (84.1%)	47 (13.9%)	4 (1.2%)
Tongue	209 (61.7%)	124 (36.6%)	3 (0.9%)
Saliva	248 (73.2%)	87 (25.7%)	1 (0.3%)
Gums and oral tissue	251 (74.0%)	72 (21.2%)	11 (3.2%)
Natural teeth	130 (38.3%)	130 (38.3%)	71 (20.9%)
Cleanliness	207 (61.1%)	`87 (25.7%)	40 (11.8%)
Dentures	145 (42.8%)	24 (7.1%)	4 (1.2%)
Dental pain	310 (91.4%)	22 (6.5%)	2 (0.6%)

n_number of participants

Score 0: *Healthy*; Score 1:*Changes*; Score 2:*Unhealthy*

^*^Individual categories do not add up to total sample size as some participants had missing data

**Table 3:** Distribution of oral health conditions and dentist visits for participants at Wave 6 (N = 339)

Variables	N	Mean(± SD)/n(%)	Range
OHAT total score^*^	336	2.5 (± 2.1)	0–11
SRSpH	337		4.5–7.8
> 6.6		280 (83.0%)	
≤ 6.6		57 (16.9%)	
Functional pairs of occluding teeth	336		0–16
> 10 pairs		127 (37.8%)	
≤ 10 pairs		209 (62.2%)	
Dental services utilization	333		
< 12 months ago		229 (68.7%)	
1–2 years ago		62 (18.6%)	
3–4 years ago		19 (5.7%)	
5–6 years ago		6 (1.8%)	
> 6 years ago		17 (5.1%)	

Individual oral health variables and dental care utilization do not add up to total sample size *(N* = *339)* as some participants had missing data

*n* number of participants, *SD* Standard Deviation, *OHAT* Oral Health Assessment Tool

SRSpH_Sub-lingual Resting Saliva pH

*Higher OHAT scores indicate poorer oral health and vice-versa

### Cognitive function and oral health

#### Oral health assessment tool (OHAT)

Lower scores in the specific cognitive domain of attention/processing speed at Wave 4, and for change in cognitive function (between Wave 1 and Wave 4) were negatively associated with OHAT scores at Wave 6 (*p*s = 0.028 and 0.024, respectively). This means that older adults with poorer attention/processing speed and who showed greater decline in this cognitive capacity over a six-year period (from Wave 1 to Wave 4) had more oral health problems four years later (Wave 6), after accounting for age, gender, and education. This association also remained robust when adjusted for comorbidities (see Additional file 1). However, after full adjustment for covariates neither of these associations remained significant. For the other four cognitive domains, as well as the composite global cognition measure, none of the models was significant after either partial or full adjustments for covariates were applied. These data are presented in Table 4.

**Table 4:** Ordinary least squares regression estimating effect of cognitive function on Oral Health Assessment Tool (OHAT) score

	Partially adjusted^a^		Fully adjusted^b^	
	B (lower, upper 95% CI)	*p* value	B (lower, upper 95% CI)	*P *value
Wave 4				
Composite global cognition	− 0.11 (− 0.35, 0.12)	0.353	0.06 (− 0.17, 0.31)	0.592
Attention/processing speed	− 0.26 (− 0.49, − 0.02)	0.028^*^	− 0.10 (− 0.34, 0.13)	0.379
Language	− 0.02 (− 0.24, 0.19)	0.812	0.05 (− 0.16, 0.28)	0.602
Executive function	− 0.03 (− 0.24, 0.17)	0.737	0.08 (− 0.12, 0.29)	0.425
Visuo-spatial	− 0.09 (− 0.32, 0.14)	0.444	− 0.00 (− 0.23, 0.21)	0.952
Memory	0.11 (− 0.13, 0.36)	0.359	0.22 (− 0.02, 0.46)	0.074
Change in cognitive function^				
Composite global cognition	− 0.12 (− 0.52, 0.27)	0.534	− 0.02 (− 0.41, 0.36)	0.910
Attention/processing speed	− 0.34 (− 0.64, − 0.04)	0.024^*^	− 0.25 (− 0.55, 0.05)	0.103
Language	0.03 (− 0.29, 0.36)	0.845	0.08 (− 0.23, 0.40)	0.611
Executive function	− 0.18 (− 0.49, 0.13)	0.262	− 0.11 (− 0.42, 0.20)	0.489
Visuo-spatial	0.17 (− 0.13, 0.47)	0.275	0.24 (− 0.05, 0.54)	0.113
Memory	− 0.05 (− 0.37, 0.27)	0.765	− 0.07 (− 0.39, 0.24)	0.634

B represents the number of point change in total OHAT score per 1 unit increase in standardised cognition score

*CI* Confidence Interval

^*^p ≤ .05, ^**^p < .01, ^***^p < .001

^a^Adjusted for age, gender and years of education

^b^Adjusted for age, gender, years of education, medical conditions, general health, depression, smoking, alcohol consumption, functionality and dental care utilization

^^^Change in cognitive function = Score at Wave 4—Scores at Wave 1 (baseline)

#### Sub-lingual resting saliva pH (SRSpH)

For SRSpH, in both the partially (OR 0.65, 95% CI: 0.42–1.00) and fully adjusted models (OR 0.63, 95% CI: 0.40–0.99), greater decline in memory function over the six-year period between Wave 1 and Wave 4 was predictive of lower SRSpH four years later at Wave 6 (*p*s = 0.050 and 0.046, respectively). This indicated that participants who experienced greater decline in memory during an earlier time point were susceptible to greater risk of acidic saliva four years later. No other significant associations emerged between cognitive function and SRSpH. These data are presented in Table 5.

**Table 5:** Binary logistic regression estimating effect of cognitive function on sub-lingual resting saliva pH (SRSpH)

	Partially adjusted^a^		Fully adjusted^b^	
	Odds Ratio (lower, upper 95% CI)	*p *value	Odds Ratio (lower, upper 95% CI)	*p *value
Wave 4				
Composite global cognition	0.92 (0.68, 1.24)	0.600	0.88 (0.63, 1.22)	0.454
Attention/processing speed	1.09 (0.80, 1.49)	0.569	1.08 (0.76, 1.52)	0.655
Language	0.84 (0.64, 1.11)	0.225	0.79 (0.58, 1.08)	0.151
Executive function	0.99 (0.76, 1.30)	0.983	0.97 (0.73, 1.30)	0.874
Visuo-spatial	0.91 (0.68, 1.23)	0.577	0.93 (0.68, 1.28)	0.681
Memory	0.81 (0.59, 1.11)	0.197	0.75 (0.52, 1.06)	0.108
Change in cognitive function^^^				
Composite global cognition	0.87 (0.53, 1.42)	0.578	0.84 (0.50, 1.42)	0.537
Attention/processing speed	1.08 (0.73, 1.59)	0.686	1.17 (0.77, 1.79)	0.452
Language	0.97 (0.64, 1.47)	0.901	0.93 (0.59, 1.48)	0.782
Executive function	0.81 (0.55, 1.20)	0.308	0.80 (0.53, 1.21)	0.294
Visuo-spatial	0.94 (0.64, 1.39)	0.790	0.96 (0.63, 1.46)	0.869
Memory	0.65 (0.42, 1.00)	0.050^*^	0.63 (0.40, 0.99)	0.046^*^

If Odds Ratio (OR), OR > 1, (OR-1)*100% represents percentage increase in the odds of having acidic saliva pH per unit *increase* in standardised cognition score

If OR < 1, ((1/OR)-1)*100% represents percentage increase in the odds of having acidic saliva pH per unit *decrease* in standardised cognition score

CI_Confidence Interval

^a^Adjusted for age, gender and years of education

^b^Adjusted for age, gender, years of education, medical conditions, general health, depression, smoking, alcohol consumption, functionality and dental care utilization

^^^Change in cognitive function = Score at Wave 4—Scores at Wave 1 (baseline)

^*^p ≤ .05, ^**^p < .01, ^***^p < .001

#### Functional occluding pairs of natural teeth

No associations were found with functional pairs of teeth, either when looking at Wave 4 cognitive function or for degree of decline (from Wave 1 to Wave 4), between global cognition or any of the specific cognitive domains in either the partially or fully adjusted models (Table 6).

**Table 6:** Binomial logistic regression estimating effect of cognitive function on functional pairs of teeth

	Partially adjusted^a^		Fully adjusted^b^	
	Odds Ratio (lower, upper 95% CI)	*p* value	Odds Ratio (lower, upper 95% CI)	*P *value
Wave 4				
Composite global cognition	1.09 (0.85, 1.38)	0.482	1.11 (0.85, 1.45)	0.432
Attention/processing speed	1.03 (0.82, 1.30)	0.762	1.07 (0.83, 1.39)	0.581
Language	1.03 (0.83, 1.29)	0.751	1.03 (0.81, 1.32)	0.757
Executive function	1.09 (0.88, 1.35)	0.413	1.12 (0.88, 1.42)	0.335
Visuo-spatial	1.10 (0.87, 1.38)	0.412	1.12 (0.88, 1.43)	0.335
Memory	1.06 (0.83, 1.35)	0.623	1.05 (0.80, 1.37)	0.699
Change in cognitive function^^^				
Composite global cognition	0.96 (0.64, 1.42)	0.843	1.02 (0.66, 1.56)	0.929
Attention/processing speed	0.96 (0.72, 1.30)	0.830	1.00 (0.72, 1.39)	0.976
Language	0.84 (0.61, 1.17)	0.314	0.87 (0.61, 1.24)	0.456
Executive function	1.29 (0.93, 1.79)	0.118	1.34 (0.94, 1.91)	0.100
Visuo-spatial	0.89 (0.66, 1.21)	0.479	0.95 (0.69, 1.31)	0.775
Memory	1.01 (0.73, 1.39)	0.940	1.06 (0.75, 1.50)	0.729

If Odds Ratio (OR), **OR > 1**, (OR-1)*100% represents percentage increase in the odds of having 10 or more functional pairs of teeth per unit *increase* in standardised cognition score

If **OR < 1**, ((1/OR)-1)*100% represents percentage increase in the odds of having 10 or more functional pairs of teeth per unit *decrease* in standardised cognition score

*CI* Confidence Interval

^a^Adjusted for age, gender and years of education

^b^Adjusted for age, gender, years of education, medical conditions, general health, depression, smoking, alcohol consumption,

functionality and dental care utilization

^^^Change in cognitive function = Score at Wave 4—Scores at Wave 1 (baseline)

## Discussion

The present study examined whether cognitive function prospectively predicted oral health outcomes in a cohort of older Australian adults. The key findings to emerge were older adults with declining memory function at an earlier point in life were then also found to be more likely to experience poor oral health when this was evaluated at a later time-point, four years later. Additionally, a six-year decline in memory function was a significant predictor of lower sublingual resting salivary pH levels four years later, with this association remaining robust in both the partially and fully adjusted models. However, no other relationships were identified between the other cognitive measures and oral health (OHAT & functional pairs of teeth).

Despite extensive literature focused on the relationship between oral health and cognitive function (PubMed identifies almost 300 hits to the search terms “oral health” AND “cognition”), and growing evidence showing that such a relationship exists, surprisingly little is understood about the temporality of this association. In particular, as noted previously, only six studies to date have used longitudinal research methods to test whether older adults’ poorer cognitive function at an earlier time point is predictive of their oral health at a later one. Moreover, in the only study to date to conduct such an assessment that included both a large sample size and an adequate assessment of cognitive function, oral health was indexed solely via self-report [23]. Thus, although Kang et al.’s [23] study provided the most compelling evidence to date that cognitive decline is predictive of later oral health deterioration, given the limitations inherent to self-report methods of oral health [24, 25], the current study adds meaningfully to the current literature by additionally including comprehensive objective and quantifiable indicators of oral health status.

The results partially align with the findings from Kang et al. [23] study, in showing that in a group of healthy, community-dwelling older adults, greater deterioration in memory function at an earlier timepoint was associated with an indicator of poorer oral health four years later. Using data from the English Longitudinal Study of Ageing (ELSA), Kang et al. found that poorer cognitive function at baseline was predictive of self-reported tooth loss approximately 12 years later [23]. However, our findings also extend these findings in a meaningful way. This is because our data suggest that this relationship between cognition decline and later oral health may be particularly driven by decline in specific cognitive domain(s).

While a methodological strength of Kang et al.’s study was the use of assessments from three domains known to be particularly sensitive to age-related cognitive decline (memory, executive control and processing speed), performance in these three domains were not considered separately, but instead used to create an aggregate measure of general cognitive function. As Kang et al. rightly note, there is considerable validity and appeal to such an approach [23]. However, in light of recent evidence that the relationship between oral health and cognition may vary across different cognitive domains [12], it was considered important to assess specific cognitive domains separately in the current study.

The current data found that a six-year decline in memory was associated with lower sub-lingual resting saliva pH levels four years later, even following adjustment for an extensive array of potential covariates. Because salivary pH plays a critical role in the maintenance of good oral health, this finding is potentially important. Changes in levels of salivary pH are observed to directly affect the process of tooth remineralization in response to an acid exposure/attack in the oral cavity. Evidence also indicates that prolonged exposure to acidic saliva (pH < 6.6) leads to demineralization of teeth [44]. The inability to maintain one’s own oral care due to increasing physical and cognitive impairment results in very poor oral hygiene and an increase in aciduric pathogenic biofilm throughout the mouth. The combination of poor oral hygiene and poor saliva quality with lower pH often leads to multiple rapidly progressing decaying teeth, some with pulpal exposures causing infections in bone that add to the inflammatory bioburden of the individual. Indeed, it has recently been argued that salivary pH should be regarded as a “quick, chairside diagnostic, biomarker of oral health status” [45].

While no prior study involving older adults has tested whether cognitive decline predicts later salivary pH specifically, recent studies have identified links between age-related cognitive decline and other aspects of salivary gland function. Sorensen et al. [46] identified hyposalivation as a correlate of age-related cognitive decline in middle-aged males, while Do et al. [47] showed that salivary flow rate was independently associated with cognitive impairment among older adults, even after controlling for various confounders. It has been suggested that the central autonomic control pathways that regulate salivary gland function might be affected by the same degenerative processes that contribute to reduced cognitive function [46]. One of the central ways in which salivary gland dysfunction may present is via disturbances in the salivary buffer system, which is responsible for maintaining salivary pH at a relatively constant level. Because salivary gland hypofunction is associated with low pH conditions in the oral environment [48], decline in higher level brain systems might therefore also present as lower pH. Future work is now needed to test this possibility. However, cognitive decline has also been linked to altered patterns of appetite and eating disturbances [49]. Because these altered patterns of eating often include an increase in cravings for unhealthy foods [50], this might also explain why cognitive status is related to lower pH, as diet quality has been shown to be an important determinant of salivary pH [51]. Thus, there are a number of potential biological mechanisms that might explain why a relationship between cognitive decline and salivary pH exists.

Finally, the current study had various strengths, including a longitudinal design, a well-characterized cohort of older adults, and the ability to control for several confounding factors. In addition, a comprehensive neurocognitive battery allowed detailed assessment of different cognitive domains and the use of multiple tests within each cognitive domain minimised the potential influence of test-specific factors. In addition, this study used multiple well validated tools (OHAT) and objective oral health indicators (e.g., SRSpH, the number of occluding pairs), rather than using subjective indicators (self-report) of oral health.

These positive features noted, it is important to acknowledge the reasons why most of the relationships assessed were not significant. One reason could be attributed to the relatively short four-year follow-up period; it would seem likely that more extended delays between baseline and follow up would have greater sensitivity to any effects of cognitive decline on subsequent oral health. However, this relatively short follow up also makes the finding of a significant association between level of memory decline with an objective indicator of oral health status more striking, and as noted, it is hoped that this specific finding (particularly in light of converging evidence from a large longitudinal study focused on middle-aged adults) will encourage further assessment of this specific indicator in understanding how oral health and cognitive function might be related. Another factor which could have meant that the relationship between cognitive function and oral health was underestimated in the current study was that the older individuals sampled were generally healthy and reported good levels of oral health. Further, limitations of the present study include the absence of detailed neuropsychological data at Wave 6 and oral health information at baseline. We also do not know if their oral health was good or poor at baseline. This clearly limits any temporal inferences that can be made, and to provide a direct test of direction of temporality, future research is needed that incorporates baseline oral health data. Moreover, future studies are now also required to understand whether this association between salivary pH and memory is robust, and if so, the underlying mechanism. If so, these findings could have implications for policy makers and health care providers to help develop and implement targeted interventions to improve oral health outcomes and quality of life in older adults with deteriorating memory function.

## Conclusion

To conclude, the key finding to emerge from this longitudinal cohort study involving 339 older adults was that memory decline over a six-year period (but no other aspect of cognitive function) predicted lower sub-lingual resting salivary pH four years later. Because lower salivary pH is considered to be an objective indicator of oral health, these data provide further important insights into our understanding of exactly when and how oral health and cognitive function may be linked in late adulthood. However, the findings of the present study must be interpreted cautiously given the cross-sectional nature of oral health data. In particular, findings from this study suggests that older adults who present with greater than normal memory decline may also be at risk of poorer oral health.

## Supplementary Information

**Additional file 1: Table S1.** Z-scores for global cognitive function and five cognitive domains. **Table S2. **Additional analyses estimating effect of cognitive function on Oral Health Assessment Tool (OHAT) score controlling for different covariates. **Figure S1.** Directed acyclic graphs (DAG) informing casual relationships between cognition and oral health. **Table S3. **Distribution of oral health variables based on OHAT score.

## Data Availability

The data that support the findings of this study are available on request from the corresponding author. The data are not publicly available due to privacy or ethical restrictions.

## Abbreviations

MAS: Memory and ageing study
MMSE: Mini mental state examination
OHAT: Oral health assessment tool
SRSpH: Sub-lingual resting salivary pH
RAVLT: Rey auditory visual verbal learning test
BVRT: Benton visual retention test
SD: Standard deviation
GDS: Geriatric depression scale
B-ADL: Bayer activities of daily living
ADL: Activities of daily living
