# Supplementary material for: Memory decline in older individuals predicts an objective indicator of oral health: findings from the Sydney Memory and Ageing Study
Source: BMC Oral Health. 2022 Mar 27;22:93. doi: 10.1186/s12903-022-02128-y (PMC8962025; doi:10.1186/s12903-022-02128-y)
Supplement: Supplementary file 1 — Additional file 1: Table S1. Z-scores for global cognitive function and five cognitive domains. Table S2. Additional analyses estimating effect of cognitive function on Oral Health Assessment Tool (OHAT) score controlling for different covariates. Figure S1. Directed acyclic graphs (DAG) informing casual relationships between cognition and oral health. Table S3. Distribution of oral health variables based on OHAT score. [file 12903_2022_2128_MOESM1_ESM.docx]

**Additional file 1**:

Table S1. Z-scores for global cognitive function and five cognitive domains.

Table S2. Additional analyses estimating effect of cognitive function on Oral Health Assessment Tool (OHAT) score controlling for different covariates

Figure S1. Directed acyclic graphs (DAG) informing casual relationships between cognition and oral health

Table S3. Distribution of oral health variables based on OHAT score.

**Table S1 Comparison of mean (standard deviation) z-scores for global function and five cognitive domains for participants from Wave 1 to Wave 4**

| **Cognitive measure** | **Wave 1** | **Wave 4** | **Mean change** | ***t* value** | ***p* value^*^** |
| --- | --- | --- | --- | --- | --- |
| Composite Global Cognition | 0.25 (0.95) | -0.08 (1.07) | -0.33 (0.59) | 10.37 | <.001 |
| Attention/Processing Speed | 0.18 (0.94) | -0.16 (1.03) | -0.37 (0.78) | 8.56 | <.001 |
| Language | 0.14 (1.02) | -0.10 (1.07) | -0.24 (0.70) | 6.29 | <.001 |
| Executive Function | 0.16 (0.96) | -0.06 (1.16) | -0.25 (0.74) | 6.11 | <.001 |
| Visuo-spatial | 0.19 (1.02) | 0.02 (1.05) | -0.18 (0.75) | 4.41 | <.001 |
| Memory | 0.28 (0.96) | 0.14 (1.03) | -0.15 (0.70) | 3.86 | <.001 |

**Paired t-test*

**Table S2 Ordinary least squares regression estimating effect of cognitive function on Oral Health Assessment Tool (OHAT) score**

|  | **Model 1^a^** | | **Model 2^b^** | | **Model 3^c^** | | **Model 4^d^** | | |
| --- | --- | --- | --- | --- | --- | --- | --- | --- | --- |
|  | **B (lower, upper 95% CI)** | ***p* value** | **B (lower, upper 95% CI)** | ***p value*** | **B (lower, upper 95% CI)** | ***p value*** | **B (lower, upper 95% CI)** | ***p value*** |  |
| **Wave 4** |  |  |  |  |  |  |  |  |  |
| Composite Global Cognition | -0.11 (-0.35, 0.12) | 0.353 | -0.02 (-0.26, 0.21) | 0.840 | -0.18 (-0.26, 0.22) | 0.883 | -0.08 (-0.3, 0.16) | 0.512 |  |
| Attention/Processing Speed | -0.26 (-0.49, -0.02) | 0.028^*^ | -0.20 (-0.43, 0.03) | 0.088 | -0.20 (-0.44, 0.03) | 0.091 | -0.20 (-0.43, 0.03) | 0.094 |  |
| Language | -0.02 (-0.24, 0.19) | 0.812 | -0.01 (-0.24, 0.20) | 0.864 | 0.07 (-0.15, 0.29) | 0.521 | -0.02 (-0.24, 0.19) | 0.828 |  |
| Executive Function | -0.03 (-0.24, 0.17) | 0.737 | 0.03 (-0.17, 0.25) | 0.718 | 0.00 (-0.20, 0.21) | 0.942 | -0.01 (-0.22, 0.19) | 0.897 |  |
| Visuo-spatial | -0.09 (-0.32, 0.14) | 0.444 | -0.04 (-0.27, 0.18) | 0.728 | -0.06 (-0.29, 0.16) | 0.580 | -0.06 (-0.29, 0.16) | 0.577 |  |
| Memory | 0.11 (-0.13, 0.36) | 0.359 | 0.20 (-0.04, 0.44) | 0.112 | 0.17 (-0.07, 0.41) | 0.164 | 0.11 (-0.13, 0.36) | 0.356 |  |
| **Change in Cognitive Function^^^** |  |  |  |  |  |  |  |  |  |
| Composite Global Cognition | -0.12 (-0.52, 0.27) | 0.534 | -0.08 (-0.48, 0.30) | 0.654 | -0.09 (-0.49, 0.29) | 0.628 | -0.05 (-0.44, 0.34) | 0.803 |  |
| Attention/Processing Speed | -0.34 (-0.64, -0.04) | 0.024^*^ | -0.36 (-0.65, -0.06) | 0.016^*^ | -0.29 (-0.59, 0.00) | 0.052 | -0.25 (-0.55, 0.04) | 0.099 |  |
| Language | 0.03 (-0.29, 0.36) | 0.845 | 0.04 (-0.27, 0.37) | 0.772 | 0.10 (-0.21, 0.43) | 0.518 | 0.00 (-0.31, 0.33) | 0.963 |  |
| Executive Function | -0.18 (-0.49, 0.13) | 0.262 | -0.11 (-0.42, 0.20) | 0.489 | -0.21 (-0.52, 0.09) | 0.169 | -0.11 (-0.43, 0.19) | 0.460 |  |
| Visuo-spatial | 0.17 (-0.13, 0.47) | 0.275 | 0.17 (-0.12, 0.48) | 0.251 | 0.20 (-0.10, 0.50) | 0.192 | 0.21 (-0.08, 0.51) | 0.165 |  |
| Memory | -0.05 (-0.37, 0.27) | 0.765 | -0.06 (-0.38, 0.25) | 0.673 | -0.07 (-0.39, 0.25) | 0.663 | -0.02 (-0.35, 0.29) | 0.858 |  |

Abbreviation: CI_Confidence Interval.

1. **^*^***p ≤*.05, ^**^p<.01, ^***^p<.001

2.^^^Change in cognitive function = Score at Wave 4 - Scores at Wave 1 (baseline)

3. B represents the number of point change in total OHAT score per 1 unit increase in standardised cognition score

4**.** ^a^Model 1 - Adjusted for age, gender and years of education.

5. ^b^Model 2 - Adjusted for age, gender, years of education + comorbidities (includes medical conditions, general health and depression)

6. ^c^Model 3 - Adjusted for age, gender, years of education + lifestyle (includes smoking history, alcohol consumption and dental care utilization)

7. ^d^Model 4 - Adjusted for age, gender, years of education + function (includes impairments in ADL and IADL)

**Figure S1 Directed Acyclic Graph (DAG) showing significant causal relationships between cognition (exposure), oral health (outcome) and covariates using data from the Sydney Memory and Ageing Study (MAS)**


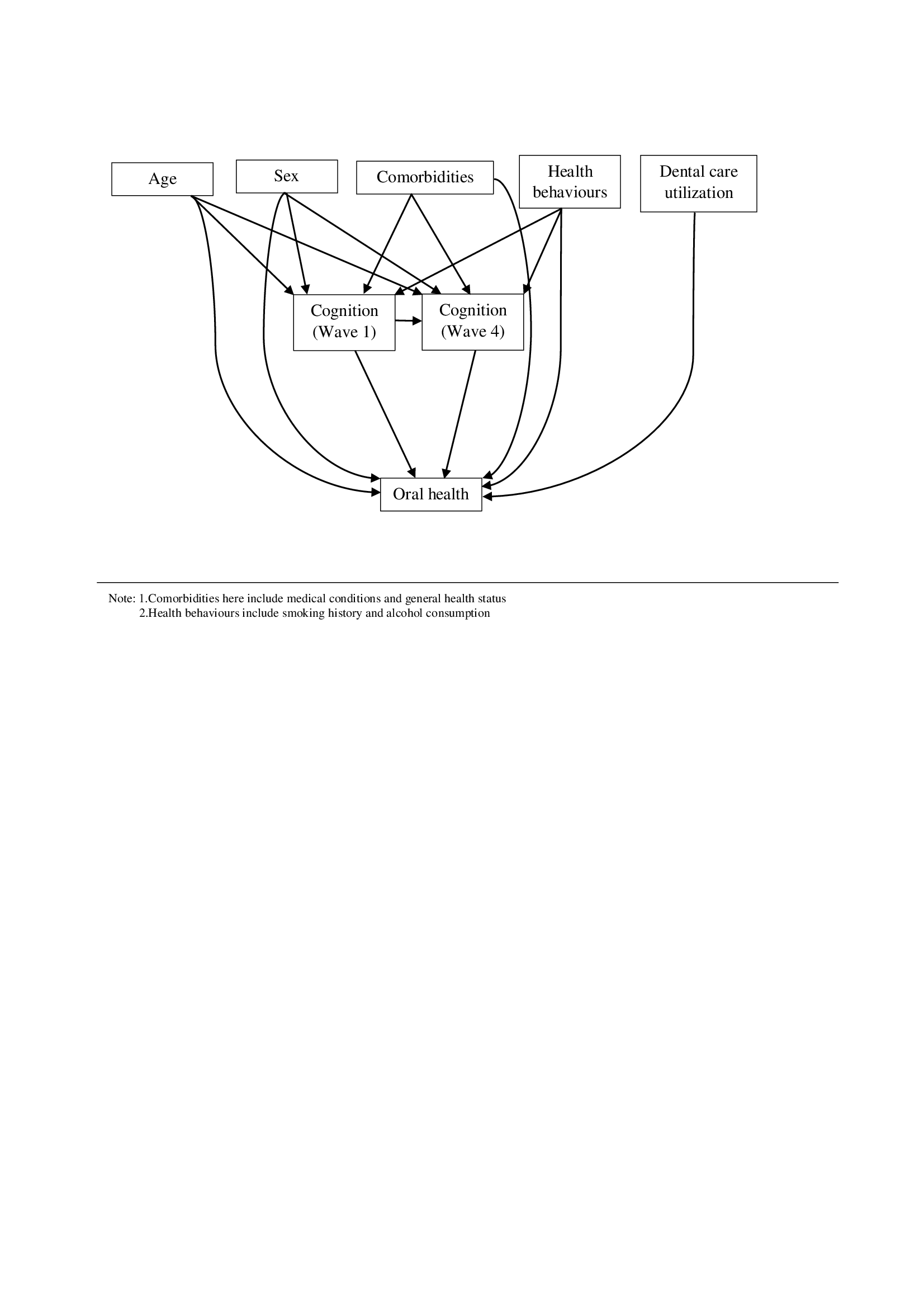


**Table S3 Distribution of oral health conditions (SRSpH and functional pairs of teeth) according to OHAT scores for participants at Wave 6 (N= 339)**

|  | **OHAT** | | | |
| --- | --- | --- | --- | --- |
|  | **0-3** | **4-7** | **8-11** | **12-16** |
|  | *n (%)* | *n (%)* | *n (%)* | *n* *(%)* |
| **SRSpH** |  |  |  |  |
| >6.6 | 206 (74.1) | 65 (23.4) | 7 (2.5) | - |
| ≤6.6 | 37 (64.9) | 19 (33.3) | 1 (1.8) | - |
| **Functional pairs of teeth** |  |  |  |  |
| >10 pairs | 93 (73.8) | 29 (23) | 4 (3.2) | - |
| ≤10 pairs | 149 (71.6) | 55 (26.4) | 4 (2) | - |

Abbreviations: OHAT_Oral Health Assessment Tool; SRSpH_Sub-lingual Resting Saliva pH

^#^Total score on OHAT ranges from 0-16. Scores on the OHAT were categorized into 4 as :

0-3=Healthy; 4-7=Mildly healthy requiring oral attention; 8-11=Unhealthy, requiring care; 12-16=Unhealthy,
